# Clomiphene Citrate Medication for Infertility and Risk of Stillbirth or Neonatal Death: A Population-based Cohort Study

**DOI:** 10.1210/clinem/dgae741

**Published:** 2024-10-22

**Authors:** Vivienne Moore, Alice Rumbold, Renae Fernandez, Heather McElroy, Lynette Moore, Lynne Giles, Luke Grzeskowiak, Elizabeth Roughead, Michael Stark, Darryl Russell, Michael Davies

**Affiliations:** School of Public Health, The University of Adelaide, Adelaide, South Australia, SA 5005, Australia; Robinson Research Institute, The University of Adelaide, Adelaide, South Australia, SA 5005, Australia; Robinson Research Institute, The University of Adelaide, Adelaide, South Australia, SA 5005, Australia; South Australian Health and Medical Research Institute, Adelaide, South Australia, SA 5005, Australia; Robinson Research Institute, The University of Adelaide, Adelaide, South Australia, SA 5005, Australia; Adelaide Medical School, The University of Adelaide, Adelaide, South Australia, SA 5005, Australia; Adelaide Medical School, The University of Adelaide, Adelaide, South Australia, SA 5005, Australia; Adelaide Medical School, The University of Adelaide, Adelaide, South Australia, SA 5005, Australia; SA Pathology, Women's and Children's Hospital, Adelaide, South Australia, SA 5006, Australia; School of Public Health, The University of Adelaide, Adelaide, South Australia, SA 5005, Australia; Robinson Research Institute, The University of Adelaide, Adelaide, South Australia, SA 5005, Australia; South Australian Health and Medical Research Institute, Adelaide, South Australia, SA 5005, Australia; College of Medicine and Public Health, Flinders University, Adelaide, South Australia, SA 5042, Australia; Quality Use of Medicines and Pharmacy Research Centre, Clinical and Health Sciences, University of South Australia, Adelaide, South Australia, SA 5005, Australia; Robinson Research Institute, The University of Adelaide, Adelaide, South Australia, SA 5005, Australia; Adelaide Medical School, The University of Adelaide, Adelaide, South Australia, SA 5005, Australia; Robinson Research Institute, The University of Adelaide, Adelaide, South Australia, SA 5005, Australia; School of Biomedicine, The University of Adelaide, Adelaide, South Australia, SA 5005, Australia; Robinson Research Institute, The University of Adelaide, Adelaide, South Australia, SA 5005, Australia; Adelaide Medical School, The University of Adelaide, Adelaide, South Australia, SA 5005, Australia

**Keywords:** polycystic ovary syndrome, clomiphene citrate, stillbirth, neonatal death, perinatal death

## Abstract

**Objective:**

To assess associations between clomiphene citrate (CC) use and perinatal death.

**Design:**

Whole of population data linkage cohort.

**Setting:**

South Australia.

**Participants:**

All women giving birth between July 2003 and December 2015 (n = 242,077).

**Methods:**

All births of at least 20 weeks were linked to government records of dispensed medications. A pregnancy was considered exposed to CC if a prescription was dispensed from 90 days before through to the end of a conception window. Descriptive statistics for stillbirths and neonatal deaths were stratified by multiplicity. For singletons, multivariable logistic regression models were used to examine the association of CC exposure with the combined outcome of perinatal death.

**Main outcome measures:**

Stillbirths and neonatal deaths (with 28 days of birth) combined as perinatal deaths.

**Results:**

Among singletons, the prevalence of stillbirth was 6.6 per 1000 births, with neonatal deaths of 2.1 per 1000 live births. Among singletons conceived with CC, stillbirth and neonatal death had a prevalence of 10.2 and 3.1 per 1000, respectively. For the combined outcome of perinatal death, the odds ratio was 1.54 (95% confidence interval 1.15, 2.07), stable upon adjustment for factors conveying biological (eg, obesity, pregestational diabetes) and social (eg, disadvantage) risks for perinatal death.

**Conclusion:**

Risk of perinatal death may be increased in pregnancies that follow use of CC. While established confounding factors related to infertility were taken into account, there may be some residual contribution of underlying infertility.

Stillbirths and neonatal deaths are a source of grief and distress for parents and can have enduring consequences for the health and well-being of families (1-3). Many of these deaths have antecedents of compromised fetal growth and poor placental function, but identifying and managing these high-risk pregnancies remains challenging (4, 5). Understanding other risk factors could improve risk prediction (6) and could provide earlier prevention opportunities, including before pregnancy (7, 8).

Treatment for infertility using procedures involving in vitro fertilization may be associated with increased risks of stillbirth and neonatal death (7, 9, 10). Interpretation of this finding is complex as factors related to infertility disorders *per se* may contribute to poor perinatal outcomes (11). Treatment for infertility can also consist of medication alone. These births lack visibility because they are incompletely captured or not recorded at all in registries of births arising from assisted reproductive technology (ART) (12). In Australia, for example, reporting is not required when medical treatment for infertility occurs outside of specialist ART clinics. However, such treatment accounts for a quarter of births conceived with all forms of medical assistance (13).

Globally, the medication most commonly used for infertility treatment outside of a specialist ART clinic is clomiphene citrate (CC). For example, in the US Nurses’ Health Study II, around 80% of women who reported infertility in the period 2000-2011 had used CC, usually as the first (and often the only) treatment (14). In Australia, CC is the only oral medication licensed for use for anovulatory infertility. Until 2023, it was a recommended first-line treatment for polycystic ovary syndrome (PCOS), now second line (15, 16).

CC is a selective estrogen receptor modulator (17). The most obvious and well-documented adverse outcome of CC use is multiple pregnancy; this can be minimized by following the recommendation for ultrasound in at least the first cycle of treatment to determine the number of dominant follicles (eg, 18). Beyond that, questions about the safety of CC for the fetus are unresolved (19), with little research on possible adverse perinatal outcomes of CC, such as perinatal death.

Infertility treatment continues to change as evidence accumulates. For instance, CC is no longer recommended as an isolated treatment for unexplained infertility (20, 21). However, clinicians in the UK have reported that they continue to prescribe CC under pressure from couples preferring treatment to expectant management (22). Practice elsewhere is largely unknown. This highlights a need to investigate the possible adverse outcomes associated with this treatment so that potential harms are appreciated as well as benefits.

Here we investigate outcomes of pregnancies following CC by linking records of dispensed medications to a perinatal registry. The aim of this study was to quantify the fetal and neonatal death rates for births conceived in proximity to CC dispensing. For singletons, a further aim was to compare the combined perinatal death rate with that for the wider population, taking into account potential confounding factors.

## Materials and Methods

A population-based cohort study was undertaken based on births in the state of South Australia from July 2003 to December 2015. All births of at least 20 weeks’ gestation (or 400 g) must be reported to the state government, which maintains the Perinatal Registry. Almost all births in the state occur in a hospital, and reporting to the registry is standardized.

The Australian Institute of Health and Welfare undertook data linkage between South Australian Perinatal Registry records and Australian Government records of dispensed medications. Details have been described elsewhere (13).

**Figure 1.**
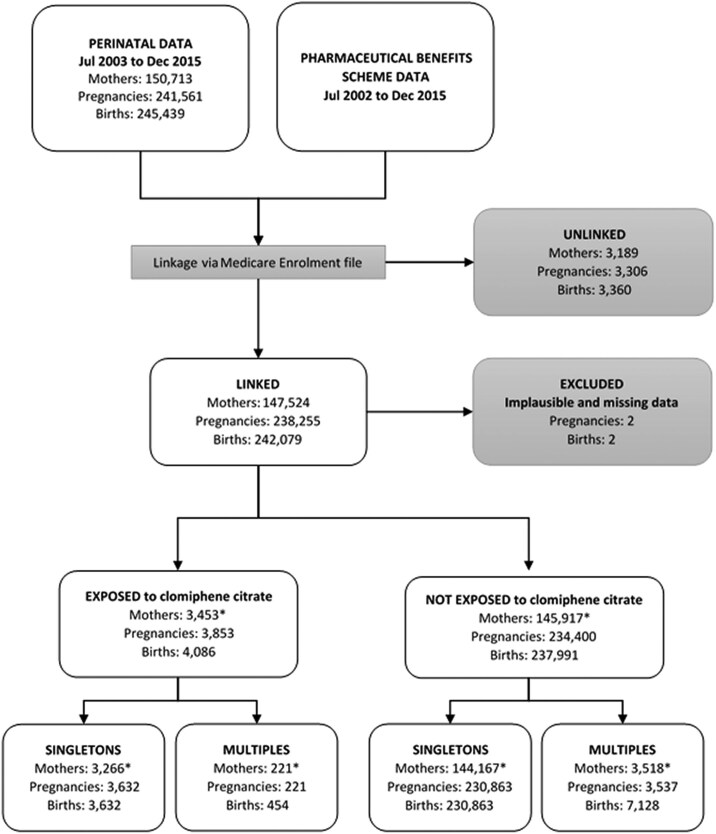
Flow diagram for data linkage. *Mothers may have more than 1 pregnancy between 2003 and 2015 and therefore numbers may sum to more than the total in the row above. Mothers may be counted in more than 1 group; for example, 1 pregnancy may have been exposed and another not exposed, or 1 pregnancy may have resulted in a singleton birth and another a multiple birth.

Briefly, the Australian Government keeps electronic records of dispensing because it subsidises the costs of approved medications. For CC, these records were complete from July 2002 apart from two 6-month periods (January to June 2009 and January to June 2012) when the cost of some brands of CC fell below the amount attracting a subsidy. Data linkage was based on identifiers of women and babies, with 2 rounds of linkage undertaken. Linkage to dispensing records was achieved for 97.9% of women (and 98.6% of pregnancies). Unlinked women and their pregnancies were excluded from the present analysis. See Fig. 1 for the flow diagram for these processes and the available data.

### Outcome

The outcomes were stillbirth after 20 weeks’ gestation and neonatal death (within 28 days), as well as the combined outcome of perinatal death. Additionally, whether the pregnancy was singleton or multiple was considered. The main focus of this analysis was singleton pregnancies; we did not consider it appropriate to combine singleton and multiple pregnancies.

### Exposure

Conception was designated as occurring proximal to CC (exposed) if a prescription for CC was dispensed from 90 days before through to the end of a preconception window (defined later). The standard quantity of medication supplied to women is 10 tablets—enough medication for 2 cycles, assuming the dose is 50 mg. We ascertained whether women were also supplied with gonadotropins in the exposure period (occasionally prescribed with CC to trigger final follicular maturation and ovulation).

It was necessary to derive a preconception window for each birth because over 20% were missing the date of last menstrual period (LMP), and births to women with anovulatory infertility may be overrepresented in this group (13). This window was based on the recorded date of birth and gestational age, with allowance for biological variation and inaccuracy of dating by routine ultrasound (13). Comparisons between available LMP dates and the derived preconception window are detailed elsewhere; where the LMP date was not missing, it was consistent with the preconception window in most cases (94.5%) (13). One missing gestational age was imputed as the 50th centile for the relevant birthweight and sex using Australian data (23).

### Covariates

**Figure 2.**
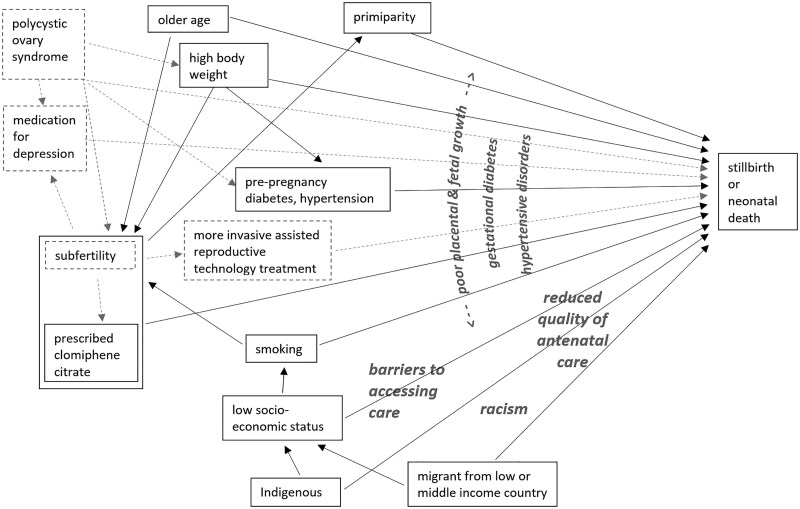
Schema illustrating our understanding of the causal structure of associations between clomiphene citrate, confounding variables, and the outcomes of stillbirth or neonatal death among singletons.

Covariate selection was based on our current understanding of casual pathways, as depicted in Fig. 2. Variables in boxes with a solid outline represent factors available for investigation while those in dashed boxes are theoretically relevant but not available. Variables not in boxes represent mediators. While previous adverse pregnancy outcomes are used to predict stillbirth and neonatal death for clinical purposes, these are not included in the diagram as they are considered to be outcomes of the underlying factors responsible for increased risk, not independent risk factors.

Maternal characteristics were obtained from the Perinatal Registry records. These included age, country/region of birth, ethnicity, pregnancy history, body mass index (BMI; collected from 2007), smoking, pre-existing hypertension, and diabetes (type 1 and type 2, not distinguished). Most of these variables were used in the format provided. However, age of each woman at the time of birth was categorized; region of birth was classified as Australia, Europe, Asia, or “other” for descriptive purposes; and in country of birth was classified as low or middle income (LMI) or high income (24). Postcode of residence was used to assign a socioeconomic Index of Relative Social Disadvantage (25), categorized in quintiles using the Australian distribution as the reference.

Certain medical conditions are known to contribute to stillbirth (26), but most of these do not entail need for CC. The exceptions are type 2 diabetes and hypertension, which are elevated in women with PCOS.

Direct contributions of PCOS to stillbirth or neonatal death are represented by a dashed line. For example, endometrial dysfunction that occurs in PCOS can affect fetal survival (27). Genetic factors that contribute to PCOS could also be responsible for compromised placental and fetal development, although this has not been established (28, 29). Other types of infertility with a genetic basis are known to affect fetal viability and survival (30-32), but in the Australian context it is unlikely that CC would be prescribed to women with conditions such as recurrent pregnancy loss or primary ovarian insufficiency.

There is some evidence of a small increase in the risk of death among male babies, especially in relation to maternal type 2 diabetes (33). Since CC does not appear to affect the ratio of male to female babies, conceptually this factor is a mediator rather than a confounder. Eight babies who died had indeterminate sex and were considered male for the purposes of this analysis.

### Statistical Analysis

We calculated the prevalence of stillbirths and neonatal deaths and tabulated data to compare characteristics of pregnancies by outcome and plurality. Some summary statistics could not be reported due to sparse data and to ensure individuals could not be reidentified.

In multivariate models for singletons, we combined the outcomes of stillbirth and neonatal death, although we acknowledge it is preferable to analyze these separately if data permits. We used logistic regression models to estimate the odds ratio (OR) for the association of exposure to CC and perinatal death, the preferred effect measure in analytic epidemiological studies (34). To account for the correlation between births to the same mother a generalized estimating equation approach with an exchangeable correlation structure was used.

A series of logistic regression models were then fit, in which different sets of adjustment covariates were sequentially added. Model 1 was based on variables in the minimal sufficient adjustment set that referenced physiological pathways (maternal age, primiparity, smoking in pregnancy, BMI, pre-existing diabetes, and pre-existing hypertension). In model 2, which represents the complete minimal adjustment set, further variables were added reflecting influences of disadvantage or discrimination (area disadvantage, Indigenous, born in LMI country). In model 3, presented for clinical interest, variables that are arguably mediators were added: the sex of the fetus as well as gestational diabetes and hypertension in pregnancy.

Missing data were generally rare, apart from mother's smoking status (n = 2791; 1.2%) and BMI (n = 98 836; 42.1%). Maternal BMI was only collected from 2007 and has gradually become more complete (51.2% missing in 2007 reducing to 8.8% in 2015).

We imputed missing values for BMI category and smoking status using multiple imputation by chained equations (35), performed in the order stated previously with fully conditional specification of the logistic regression method (cumulative logit for BMI) and 100 burn-ins before each imputation. In addition to all variables used in modeling, we included auxiliary variables of Caucasian (yes/no) and Asian (yes/no), as these were relevant to the missing at random assumption. We generated 30 imputed datasets and combined the estimates using Rubin's rules. We also generated results using the complete cases as well as using a missing indicator approach, and we explored why these are biased.

SAS version 9.4 was used in all analyses. The procedures MI and MIANALYZE were used for the multiple imputation analyses.

### Ethics Approval

Approval for the study was obtained from the ethics committees of the South Australian Department of Health [HREC/15/SAH/80] and the Australian Institute of Health and Welfare [EO2013/3/51]. Individual level consent was not required because the study entailed the use of deidentified records (managed through the Secure Unified Research Environment (SURE) facility).

## Results

There were 242 077 births of at least 20 weeks’ gestation to 147 524 women in the study period. Among singletons (96.9% of births), the prevalence of stillbirth was 6.6 per 1,000, with neonatal deaths of 2.1 per 1000 live births. Corresponding prevalences for multiples were 15.6 and 10.3 per 1000.

Maternal and pregnancy characteristics are compared in Table 1. As expected, among singletons there was an excess of stillbirths where mothers were aged 40 years or more, had not previously had an ongoing pregnancy, or were smoking at the time of the first antenatal visit. Stillbirths were also elevated where mothers were disadvantaged, or migrants from a LMI country. Similar patterns were observed for neonatal deaths. Pre-existing diabetes and hypertension in mothers were clearly associated with increased occurrence of stillbirth and neonatal death. The majority of singleton stillbirths (83%) and neonatal deaths (75%) occurred before 37 weeks of gestation.

**Table 1.** Maternal and pregnancy characteristics by perinatal outcome (births in South Australia, July 2003-December 2015), stratified by multiplicity

Characteristic	Singleton births (n = 234 495)						Multiple births (n = 7582)					
	Stillbirth (n = 1541)		Neonatal death (n = 481)		Survived > 28 days (n = 232 473)		Stillbirth (n = 118)		Neonatal death (n = 77)		Survived > 28 days (n = 7387)	
	n	%	n	%	n	%	n	%	n	%	n	%
Age (years)												
< 25	347	22.5	118	24.5	44 370	19.1	12	10.2	9	11.7	861	11.7
25 to <30	403	26.2	127	26.4	66 671	28.7	36	30.5	21	27.3	1852	25.1
30 to <35	445	28.9	124	25.8	74 956	32.2	45	38.1	32	41.6	2593	35.1
35 to <40	263	17.1	92	19.1	38 199	16.4	np	np	np	np	np	np
≥ 40	83	5.4	20	4.2	8277	3.6	np	np	np	np	np	np
Ethnicity												
Caucasian	1225	79.5	377	78.4	195 382	84.1	102	86.4	70	90.9	6517	88.2
Asian	149	9.7	49	10.2	20 156	8.7	np	np	np	np	np	np
Other	167	10.8	55	11.4	16 935	7.3	np	np	np	np	np	np
Region of birth												
Australia	1220	79.2	386	80.2	186 821	80.4	105	89.0	70	90.9	6326	85.6
Asia	160	10.4	54	11.2	22 278	9.6	np	np	np	np	np	np
Europe	76	4.9	16	3.3	11 723	5.0	np	np	np	np	np	np
Other	85	5.5	25	5.2	11 651	5.0	np	np	np	np	np	np
Low- to middle-income country of birth	227	14.7	69	14.3	30 407	13.1	13	11.0	5	6.5	617	8.3
Area index of relative socioeconomic disadvantage quintile												
1 (most disadvantaged)	463	30.1	134	27.9	60 173	25.9	28	23.7	20	26.0	1665	22.5
2	498	32.3	162	33.7	71 123	30.6	35	29.7	30	39.0	2252	30.5
3	139	9.0	54	11.2	25 265	10.9	6	5.1	6	7.8	817	11.1
4	256	16.6	86	17.9	49 304	21.2	34	28.8	14	18.2	1714	23.2
5 (least disadvantaged)	185	12.0	45	9.4	26 608	11.5	15	12.7	7	9.1	939	12.7
Tobacco smoker at first antenatal visit												
Yes	338	21.9	127	26.4	42 616	18.3	16	13.6	6	7.8	1210	16.4
No	1100	71.4	334	69.4	187 189	80.5	98	83.1	64	83.1	5900	79.9
Unknown	103	6.7	20	4.2	2668	1.2	4	3.4	7	9.1	277	3.8
Body mass index (kg/m^2^)												
Underweight and healthy weight (<25)	395	25.6	102	21.2	65 034	28.0	28	23.7	11	14.3	1574	21.3
Overweight (25 to <30)	232	15.1	77	16.0	36 979	15.9	22	18.6	10	13.0	1005	13.6
Obese (≥30)	223	14.5	65	13.5	32 552	14.0	12	10.2	15	19.5	1080	14.6
Unknown	691	44.8	237	49.3	97 908	42.1	56	47.5	41	53.3	3728	50.5
Metabolic health												
Prepregnancy diabetes	39	2.5	9	1.9	1544	0.7	—	np	np	np	np	np
Prepregnancy hypertension	29	1.9	11	2.3	2677	1.2	np	np	np	np	np	np
Obstetric history and pregnancy complications												
No previous pregnancy resulting in birth (parity = 0)	696	45.2	210	43.7	97 505	41.9	44	37.3	39	50.7	3096	41.9
Previous miscarriage	400	26.0	136	28.3	54 704	23.5	31	26.3	16	20.8	1920	26.0
Previous neonatal death	17	1.1	14	2.9	1015	0.4	Np	np	np	np	np	np
Gestational diabetes	45	2.9	23	4.8	14 559	6.3	5	4.2	5	6.5	704	9.5
Hypertension during pregnancy	88	5.7	33	6.9	17 922	7.7	Np	np	np	np	np	np
Gestation at birth (weeks)												
Under 32	1088	70.6	294	61.1	1969	0.9	86	72.9	68	88.3	759	10.3
32 to 36	184	11.9	67	13.9	13 390	5.8	18	15.3	6	7.8	3783	51.2
37 to 40	237	15.4	103	21.4	190 189	81.8	np	np	np	np	np	np
≥ 41	32	2.1	17	3.5	26 925	11.6	np	np	np	np	np	np
Male baby	800	51.9	265	55.1	119 538	51.4	56	47.5	41	53.3	3669	49.7

Abbreviations: np, not provided to protect confidentiality.

In all, there were 3853 pregnancies exposed to CC (1.6%), with 94.3% of these pregnancies being singletons. In 5.7% of the CC pregnancies, gonadotropin was also prescribed, but this did not contribute to the excess of multiples.

Singleton births that occurred after exposure to CC (Table 2) tended to involve Caucasian women and mothers over 30 years of age, with the most disadvantaged women underrepresented. The proportion of births that occurred before 37 weeks’ gestation was higher among those exposed to CC (8.7%) than in the unexposed group (7.2%).

**Table 2.** Maternal and pregnancy characteristics and perinatal outcome for singleton births (South Australia, July 2003-December 2015), according to CC exposure

Characteristic	Exposed to CC (n = 3632)		Not exposed to CC (n = 230 863)	
	n	%	n	%
Age (years)				
Under 25	277	7.6	44 558	19.3
25 to <30	1182	32.5	66 019	28.6
30 to <35	1426	39.2	74 099	32.1
35 to <40	624	17.2	37 930	16.4
≥ 40	123	3.4	8257	3.6
Ethnicity				
Caucasian	3285	90.4	193 699	83.9
Asian	228	6.3	20 126	8.7
Other	119	3.2	17 038	7.4
Region of birth				
Australia	3101	85.4	185 326	80.3
Asia	256	7.0	22 236	9.6
Europe	166	4.6	11 649	5.0
Other	109	3.0	11 652	5.0
Low- to middle-income country of birth	340	9.4	30 363	13.2
Area index of relative socioeconomic disadvantage quintile				
1 (most disadvantage)	717	19.7	60 053	26.0
2	1090	30.0	70 693	30.6
3	397	10.9	25 061	10.9
4	892	24.6	48 754	21.1
5 (least disadvantage)	536	14.8	26 302	11.4
Tobacco smoker at first antenatal visit				
Yes	274	7.5	42 807	18.5
No	3301	90.9	185 322	80.3
Unknown	57	1.6	2734	1.2
Body mass index (kg/m^2^)				
Underweight and healthy weight (<25)	815	22.4	64 716	28.0
Overweight (25-<30)	505	13.9	36 783	15.9
Obese (≥30)	597	16.4	32 243	14.0
Unknown	1715	47.2	97 121	42.1
Metabolic health				
Pre-existing diabetes	57	1.6	1535	0.7
Pre-existing hypertension	59	1.6	2658	1.2
Obstetric history and pregnancy complications				
No previous pregnancy resulting in birth	2092	57.6	96 319	30.0
Previous miscarriage	991	27.3	54 249	23.5
Previous neonatal death	23	0.6	1023	0.4
Gestational diabetes	413	11.4	14 214	6.2
Hypertension during pregnancy	383	10.5	17 660	7.6
Gestation at birth (weeks)				
Under 32	75	2.1	3276	1.4
32 to 36	241	6.6	13 400	5.8
37 to 40	3030	83.4	187 499	81.2
≥ 41	286	7.9	26 688	11.6
Male baby	1836	50.6	118 767	51.4
Perinatal outcome				
Survived neonatal period (28 days)	3584	98.7	228 889	99.1
		**Per 1000**	1504	**Per 1000**
Stillborn	37	10.2	1504	6.5
Neonatal death	11	3.1	470	2.1

Abbreviations: CC, clomiphene citrate.

Frequencies of stillbirth and neonatal death were elevated after exposure to CC (Table 2). The prevalence of stillbirth was 10.2 per 1000 births, while neonatal death occurred in 3.1 per 1000 live births.

The crude OR for the association between CC exposure and stillbirth was 1.57 [95% confidence interval (CI) 1.13, 2.18] for singleton births. The crude OR for CC exposure and neonatal death was 1.49 (95% CI 0.82, 2.71) for singleton live births.

The crude OR for CC exposure and the pooled outcome of perinatal death was 1.54 (95% CI 1.15, 2.07). This result was stable in model 1, in which adjustment was made for covariates contributing to perinatal death through biological pathways, and in model 2, in which social determinants were added, as shown in Table 3. For clinical interest, in model 3 variables that are arguably on the causal pathway were added: fetal sex, gestational diabetes, and hypertension in pregnancy. The magnitude of the OR increased in this last scenario.

**Table 3.** Adjusted odds ratios for exposure to clomiphene citrate and perinatal death (births in South Australia, July 2003-December 2015)

Model using generalized estimating equations and multiple imputation	Adjusted OR	95% CI
Model 1: adjusted for maternal age, primiparity, smoking, body mass index, pre-existing diabetes, pre-existing hypertension	1.56	1.15-2.07
Model 2: Model 1 covariates and whether or not mother was Caucasian, Indigenous, migrant from low- or middle-income country as well as residential disadvantage quintile	1.59	1.18-2.13
Model 3: Model 2 covariates and variables that may be on the causal pathway—sex of baby, gestational diabetes, hypertension in pregnancy	1.64	1.22-2.21

Abbreviations: CI, confidence interval; OR, odds ratio.

We note that if we had restricted our analysis to complete cases, we would have observed somewhat stronger associations (OR = 1.85, 95% CI 1.25, 2.74) for the equivalent of model 2. Investigation of this indicated that it was driven by a relatively high occurrence of perinatal death in pregnancies of women whose smoking status or BMI was unknown (and who were unexposed). Using a missing indicator approach yielded results that were closer to multiple imputation (OR = 1.57, 95% CI 1.16, 2.11 for the equivalent of model 2).

## Discussion

In our population-based cohort study, there was a 50% increase in the risk of singleton stillbirth or neonatal death among women who were dispensed CC around the time of conception. This was stable in models that accounted for established confounding variables. The frequency of multiple pregnancy (mostly twins) among those dispensed CC was consistent with consumption of medication. However, multiples were not included in our main analyses because their risk of adverse perinatal outcomes is substantially greater for different reasons.

To our knowledge, there are few well-designed studies assessing a possible link between CC (without other fertility treatment procedures, such as intrauterine insemination) and stillbirth or neonatal death. There are several reasons for this knowledge gap: early cohort studies were too small for differences in these outcomes to be detected (see 36); until recently, the focus of trials was on achieving clinical pregnancy, so most trials did not continue to birth (37); and in registry-based studies, information on medications used in isolation to treat infertility is rarely available (11, 38).

Fertility medications were considered in a case-control study (>300 cases) of stillbirths and deaths within 24 hours of birth, undertaken by Pastore et al (39). Among case mothers, 4.8% had taken fertility medication around the time of conception, with an adjusted OR for stillbirth of 1.9 (95% CI 0.8, 4.5). Of 26 women reporting the use of fertility medications, 20 provided the name, which for 14 was a brand of CC.

Established confounding factors included in our analyses were associated with risks of perinatal death in line with published findings (7). There is some uncertainty around the role of gestational diabetes and hypertension, each of which potentially confers a 30% to 40% increase in risk of stillbirth but may not do so when proactively identified and treated (40, 41). In our study, the apparently protective effect of these conditions probably reflects good access to high-quality antenatal care.

It is possible that CC has a direct effect on the risk of stillbirth and neonatal death. CC is a selective estrogen receptor modulator with both agonist and antagonist effects in different contexts. Concerns about safety for the fetus are longstanding, partly due to the long persistence of CC and its metabolites in the body and the potential for bioaccumulation over successive cycles (19, 42). Overlapping potency in the cytotoxic and estrogen receptor antagonist activities of CC has been reported (43). CC exposure can cause epigenetic changes (44) and DNA strand breaks (45, 46). CC is reported to cause reduced endometrial thickening (47) and has known off-target effects (44, 48, 49) that could affect placental and fetal development. Recent work has demonstrated biological plausibility of adverse pregnancy outcomes with observed dose-response relationships of clomiphene and developmental anomalies and fetal death in a mouse model (50).

It has been suggested that parental infertility contributes to adverse perinatal outcomes in offspring conceived with medical assistance. Genetic factors that underlie some types of infertility are related to fetal survival and perinatal outcomes (30, 51), as are conditions such as type 1 and type 2 diabetes (52). Research aiming to disentangle “patient” from “treatment” factors has been occurring in relation to ART (11, 53), although, to date, most investigations do not consider perinatal death.

Research examining parental and treatment influences on other perinatal outcomes after ART suggests that findings pointing to infertility *per se* as a risk factor may be inflated by a lack of data on parents’ wider health profiles. For example, Messerlian et al (54) showed that most types of infertility did not have a direct effect on risk of preterm birth after established maternal risk factors were taken into account, the exceptions being when infertility reflected uterine abnormalities (eg, fibroids), endometriosis, or tubal blockages. However, results of Duneitz et al (55) suggested preterm birth in singletons was increased with all underlying infertility diagnoses in parents as well as with each step in ART treatment. Of note, these studies differed in available data, with Duneitz et al (55) lacking maternal smoking and BMI. Health profiles of parents are thus relevant in both studies, including aspects that contribute to infertility, but the residual contribution of infertility *per se* may be small after other aspects of parental health are well characterized.

In our study, we had substantial information on maternal factors, including maternal smoking and BMI. It is unlikely that women taking CC had infertility due to uterine abnormalities, endometriosis, or tubal blockages. Most likely, women had PCOS or the infertility was unexplained; the residual risk conveyed by these conditions remains unclear. A recent study found that the risk of stillbirth in women with PCOS was increased by 50% (OR = 1.50, 95% CI 1.28, 1.77), but treatment for infertility was not explored as a contributing factor (56).

There are several factors that we did not take into account that might represent confounding variables, with evidence in relation to stillbirth emerging. Arguably, these would have a minor influence on results. For example, alcohol and cannabis may contribute to stillbirth (7, 57, 58), but women desiring pregnancy and requiring fertility treatment are highly likely to abstain from use (eg, 59). Most medications for which risks to the fetus are known, or suspected, are not likely to be differentially used by women who need CC. An exception is medication for depression, which may be relatively more common among women with PCOS (60) and those experiencing infertility (61), although the association with stillbirth is uncertain (62).

The possibility has been raised that elevated risk of adverse perinatal outcomes, in general, in children conceived with various forms of medical assistance reflects greater reluctance on the part of their parents to terminate for congenital anomalies (63). However, this does not seem to be the case in practice (64).

Turning to the implications of our findings, it has been suggested that overdiagnosis and overtreatment of infertility could be reduced if couples desiring pregnancy were encouraged to attempt to conceive naturally for at least 2 years, appropriate in many circumstances (65). For women with PCOS, lifestyle intervention, particularly directed toward reducing body weight, might improve the chance of natural conception, but greater effort is needed to generate the evidence base and practical supports for women (66, 67). Letrazole is now recommended as the first-line treatment for women with PCOS, replacing CC (16). When CC is contemplated, consideration of possible risks as well as benefits should be part of informed decision-making. Pregnancies achieved with CC, like those in women with PCOS more broadly (16), should be considered high risk with women supported and monitored.

### Strengths and Limitations

A strength of our study is the high-quality data in terms of population coverage, reporting consistency, and the high proportion of linked records. Data on terminations is of high quality in South Australia, so it is unlikely that terminations have been misclassified as stillbirths. Established risk factors for stillbirth that might inflate the association between CC exposure and perinatal death were taken into account.

We cannot be certain that the dispensed CC medication was consumed, but motivation to take this medication is high and the simple regimen was shown to facilitate adherence in a clinical trial (68). The frequency of multiple pregnancy in the group dispensed CC was consistent with widespread consumption. Any misclassification from assuming medication was consumed when that was not the case would bias results toward the null.

There were 2 periods of 6 months’ duration in the 13 years of exposure data when dispensing was underascertained. Thus there may be additional CC pregnancies, but any misclassification would lead to underestimation of the association between CC and perinatal death. Information on other forms of ART treatment was lacking, but this represents a conservative bias, since women who became pregnant by this means (with an increased risk of perinatal death) were included in the group unexposed to CC; such women would constitute only a small fraction of that group (around 4%) (69).

## Conclusion

One in every 60 births in South Australia was conceived proximal to CC dispensing during the study period. The risk of perinatal death was 50% higher in these births than in the wider population, after taking into account established risk factors for this adverse outcome, including maternal smoking and high BMI. The effect of CC on perinatal death seems unlikely to be wholly explained by parental infertility. Most medical treatments have potential harms as well as benefits, and greater appreciation of this could be used to extend the time frame in which couples attempt to conceive naturally, where appropriate.

## Data Availability

The authors do not have permission to share the data as this was provided specifically for the scope of research as approved by the ethics committees. Requests to access these datasets should be directed to the data custodians.
